# Factors associated with self-reported ill health among older Ugandans: A cross sectional study

**DOI:** 10.1016/j.archger.2015.05.006

**Published:** 2015

**Authors:** Stephen Ojiambo Wandera, Valerie Golaz, Betty Kwagala, James Ntozi

**Affiliations:** a Department of Population Studies, School of Statistics and Planning, College of Business and Management Sciences, Makerere University, P.O. Box 7062, Kampala, Uganda; b Consortium for Advanced Research Training in Africa (CARTA Cohort 2) Fellow, Program Based at the African Population and Health Research Centre (APHRC), Nairobi, Kenya; c Institut National D’etudes Demographiques (INED), 133 Boulevard Davout, Paris, France

**Keywords:** Sub-Saharan Africa, Uganda, Elderly, Health status, Self-rated health

## Abstract

**Introduction:**

There is limited research on the prevalence and factors associated with self-reported ill health among older people in Uganda.

**Objective:**

Therefore, the aim of this paper was to estimate the prevalence of self-reported ill health and to identify associated risk factors among older people (age 50+) in Uganda.

**Materials and methods:**

We conducted secondary analysis of a cross sectional survey data from a weighted sample of 2382 older persons from the 2010 Uganda National Household survey. We used frequency distributions for descriptive statistics, chi-square tests (significance set at 95%) to identify initial associations and multivariable logistic regressions reporting odds ratios to examine observed associations with self-reported ill health.

**Results:**

Over half (62%) of the older people reported ill health in the 30 days preceding the survey. Self-reported ill health was positively associated with being a woman, being among the oldest old, living in the eastern region, being a household head, being Catholic, self-reported non-communicable diseases (NCDs) and being disabled.

**Conclusion:**

Gender differentials exist in self-reported ill health among older persons in Uganda.

## Highlights

• We estimate the prevalence of self-reported ill health among older people in Uganda.
• Cross sectional national survey data of 2382 older persons is used.
• Most (62%) older Ugandans reported ill health.
• The women, oldest old, household heads, Catholics reported poorer health.
• Those with non-communicable diseases (NCDs) and disability reported poor health.

## 1 Introduction

Population aging is a global concern in the World today. Two thirds of older people (age 60+) live in developing countries including sub-Saharan Africa (UNFPA & HAI, 2012). In Uganda, the population of persons age 60 and older increased from 1.1 million in 2002 to 1.3 million in 2010 (UBOS, 2010) and is expected to increase from 1.6 million in 2014 to 5.5 million by 2050 (UN, 2013). In Uganda, the retirement age of 60 years is used to define older persons (MoGLSD, 2009). In this paper however, we defined older persons as those aged 50 years and above, as recommended by the World Health Organization (WHO) and the INDEPTH network data for African contexts (Gómez-Olivé, Thorogood, Clark, Kahn, & Tollman, 2013; Hirve, Juvekar, Lele, & Agarwal, 2010; Kyobutungi, Egondi, & Ezeh, 2010; Mwanyangala et al., 2010a; WHO, 2015).

One of the growing concerns related to population aging and health in SSA is the “vulnerability of older persons to poor health outcomes” (Aboderin, 2010) such as non-communicable diseases (NCDs), disabilities or functional limitations; and the lack of access to healthcare or age-related exclusion in access to healthcare (Aboderin, 2010; Aboderin & Ferreira, 2008; UN, 2002).

To address the health needs of older persons, international, regional, and national institutions enacted several policy frameworks. These include the 2002 Madrid International Plan of Action on Aging abbreviated as the MIPAA (UN, 2002), the African health strategy (AU, 2007) and in Uganda, the policy on older persons (MoGLSD, 2009). As result of the policy, Uganda is piloting a non-contributory scheme for older persons called social assistance grants for empowerment (SAGE) in 14 out of 112 districts. In this scheme, older persons age 65 years or 60 years in Karamoja region, receive 25,000 Uganda shillings monthly, an equivalent of $10 (MoGLSD, 2011). The Ugandan policy for older persons also recommended research on the health of older persons because of the poorly developed medical records. So far, self-reported health has been a key source of data on health needs.

Although self-rated health or self-reported health (SRH) is primarily subjective, it is useful for assessing the health of a population for which objective measures are scarce. SRH focuses on one's health status – whether it is excellent, very good, good, fair or poor (Phaswana-Mafuya, Peltzer, Chirinda, Kose, et al., 2013). SRH has also been measured using self-reports on physical functioning or disability, morbidity (including non-communicable disease) and hospitalization (Bodde, Seo, & Frey, 2009; Lee, Huang, Lee, Chen, & Lin, 2012; Phaswana-Mafuya, Peltzer, Chirinda, Kose, et al., 2013). In addition, SRH can be measured by self-reporting on the incidence of ill health or illness (Drum, Horner-Johnson, & Krahn, 2008; Kabir et al., 2003a; UBOS, 2010) in national surveys. In this paper, we used the incidence of being sick or ill during the past 30 days preceding the survey (UBOS, 2010) to measure ill health among older people.

Factors associated with SRH range from demographic to socio-economic. Advanced age was associated with poor physical health (Phaswana-Mafuya, Peltzer, Chirinda, Kose, et al., 2013; Tomás, Gutiérrez, Sancho, & Galiana, 2012; Wasiak et al., 2014) for instance in Angola (Tomás et al., 2012), self-reported NCDs in South Africa (Phaswana-Mafuya, Peltzer, Chirinda, Musekiwa, et al., 2013; Ward & Schiller, 2013), multi-morbidity in Ghana (Nimako, Baiden, Sackey, & Binka, 2013). Advanced age leads to reduced immune response against illnesses, increased mobility limitations, and NCDs (Phaswana-Mafuya, Peltzer, Chirinda, Musekiwa, et al., 2013; Ward & Schiller, 2013). However, the Ghanaian study was hospital-based and did not predict the community level prevalence. In developed countries like Sweden, the pattern is similar (Marengoni, Winblad, Karp, & Fratiglioni, 2008).

With respect to gender and aging, older women report poorer health outcomes than the men. Paradoxically, older men experience higher mortality than older women (Kakoli & Anoshua, 2008). Being a woman was associated with depression in South Africa (Nyirenda, Chatterji, Rochat, Mutevedzi, & Newell, 2013; Phaswana-Mafuya, Peltzer, Chirinda, Kose, et al., 2013) and non-communicable diseases (Khanam et al., 2011a; Marengoni et al., 2008; Phaswana-Mafuya, Peltzer, Chirinda, Musekiwa, et al., 2013; Stelmach, Kaczmarczyk-Chałas, Bielecki, Stelmach, & Drygas, 2004; Ward & Schiller, 2013), poor physical health (Phaswana-Mafuya, Peltzer, Chirinda, Kose, et al., 2013; Wasiak et al., 2014), multi-morbidity in Ghana (Nimako et al., 2013) and Bangladesh (Khanam et al., 2011b) and hospitalization. The possible explanations are that women live in unfavorable socio-economic conditions (Nimako et al., 2013). The female gender disadvantage prevails even in developed countries (Marengoni et al., 2008) except in an Australian study (Britt, Harrison, Miller, & Knox, 2008). In addition, women easily report their health challenges more than the men, because the latter are not expected to acknowledge illness as readily as women do (Kabir et al., 2003a). Women are more sensitive to their health conditions than men due to their frequent interface with the health care system earlier in life (Kakoli & Anoshua, 2008; Razzaque, Nahar, Khanam, & Streatfield, 2010).

Residing in urban areas is associated with poor SRH including NCDs in South Africa (Phaswana-Mafuya, Peltzer, Chirinda, Musekiwa, et al., 2013). Urban areas are associated with sedentary lifestyles and poor dietary habits (Hosseinpoor et al., 2012). Rural residents engage in active lifestyles which increases their physical activity, lowers the risk of obesity and hypertension and therefore improves their survival rates (Fantahun, Berhane, Hogberg, Wall, & Byass, 2009). Being not married (never married, separated and or divorced) is associated with poor health (Agrawal & Keshri, 2014; Gómez-Olivé, Thorogood, Clark, Kahn, & Tollman, 2010; Khanam et al., 2011a; Phaswana-Mafuya, Peltzer, Chirinda, Kose, et al., 2013; Razzaque et al., 2010).

Low levels of education are associated with poor health (Phaswana-Mafuya, Peltzer, Chirinda, Kose, et al., 2013), depression in Amsterdam, Netherlands (Koster et al., 2006; Stelmach et al., 2004) and NCDs in South Africa (Phaswana-Mafuya, Peltzer, Chirinda, Musekiwa, et al., 2013) and Sweden (Marengoni et al., 2008). Low education limits access to health services especially for older persons (Phaswana-Mafuya, Peltzer, Chirinda, Kose, et al., 2013). On the other hand, better education is associated with a lower prevalence of NCDs in South Africa because the more educated utilize health education or information better than the less educated (Alaba & Chola, 2013).

Low income is associated with depression among older persons in Amsterdam, Netherlands (Koster et al., 2006). High income is associated with self-reported non-communicable diseases in South Africa (Alaba & Chola, 2013; Phaswana-Mafuya, Peltzer, Chirinda, Musekiwa, et al., 2013) and Bangladesh (Khanam et al., 2011b). Affluent households in developing countries, including South Africa, are more likely to adopt a western lifestyle and diets (Alaba & Chola, 2013; Hosseinpoor et al., 2012). Disability or functional limitations have been associated with poor health in Angola (Tomás et al., 2012) and South Africa (Phaswana-Mafuya, Peltzer, Chirinda, Kose, et al., 2013).

In Uganda, studies on the health of older persons have explored disability (Wandera, Ntozi, & Kwagala, 2014), and HIV and AIDS (MRC & UVRI, 2011; Nankwanga, Phillips, & Neema, 2009). However, investigating self-reported ill health and associated factors among older people, preferably using a nationally representative sample, has received limited attention. Despite the available evidence about ill health in other developing countries, there is limited evidence on the risk factors for ill health among older persons in Uganda. Therefore, the objective of this paper was to estimate the prevalence of ill health and identify the associated risk factors, among older people in Uganda.

## 2 Materials and methods

### 2.1 Data

The study used the 2010 Uganda National Household Survey (UNHS) data. The UNHS was a cross-sectional design, which used a two-stage stratified sampling. At the first stage, 712 enumeration areas were drawn using probability proportional to size. At the second stage, households were drawn using systematic sampling. A total of 6800 households were interviewed in the survey (UBOS, 2010).

Using the variable age, we selected older persons (50+) from the sample for further analysis. We obtained unweighted and weighted samples of 2628 and 2382 older persons respectively. We applied individual weights using the survey command (svy) in STATA 13 to account for the survey design including clustering and stratification. In the results, we reported weighted analyses. The decision to select persons aged 50 years and above was based on the fact that several studies using WHO and INDEPTH network data define older persons starting at age 50 for African contexts (Gómez-Olivé et al., 2013; Hirve et al., 2010; Kyobutungi et al., 2010; Mwanyangala et al., 2010a).

### 2.2 Explanatory variables

The UNHS data covered individual and household characteristics - demographic and socio-economic characteristics, disability, health, and housing conditions. Demographic factors included: gender (male or female), age group, region, place of residence (rural or urban), living arrangement, relationship to household head, and marital status. Age was recoded into four age categories: 50–59, 60–69, 70–79 and 80+. Region was recoded into four categories (1 = central, 2 = eastern, 3 = northern and 4 = western). Living arrangements was recoded into two categories (living alone and with others). Relationship to household head was recoded into three categories (1 = head, 2 = spouse and 3 = relative). Non-relatives were only six in number and we merged them with relatives to avoid losing cases. Marital status was recoded into three categories (1 = married, 2 = separated or divorced or never married and 3 = widowed). We merged three never married older persons with the separated and divorced category in the data in order to retain the cases.

Socio-economic factors included education level, religion, household poverty status, household major source of earnings, having learnt a technical skill, ownership of bicycle. Education level was recoded into no education, primary and secondary or higher education. Religion was recoded into Catholic, Anglican, Muslim, Pentecostal and Seventh Day Adventists and others. Household poverty status was generated from household expenditures and recoded 1 = poor if a household spent less than $1 a day and 0 = not poor, if a household spent greater than $1 a day). Household major source of earnings was recoded into farming, wages and remittances. There were no older persons without a form of earnings. Having learnt a technical skill or trade and household bicycle ownership were binary variables (0 = no, 1 = yes).

Disability questions focused on six domains or indicators in the UNHS survey. The six domains included seeing, hearing, walking or climbing, remembering or concentrating, self-care and communication. Therefore, being disabled was operationalized as either (a) having a lot of difficulty on any of the six indicators; (b) being unable to perform at all on any of the six indicators; or (c) having some difficulty with at least two of the six indicators. This approach to measuring disability has been used in other studies (Braithwaite & Mont, 2009; Mitra & Sambamoorthi, 2013; Mont, 2007). A detailed description of how this variable was generated can be found elsewhere (Wandera et al., 2014). Self-reporting of diabetes, heart diseases and high blood pressure were used to estimate the prevalence of NCDs, recoded as a binary variable (UBOS, 2010).

### 2.3 Outcome variable

Health related information was collected on illnesses in the last 30 days preceding the survey, and self-reported NCDs (UBOS, 2010). All usual or regular household members were asked the following question: During the past 30 days, did you suffer from any illness or injury? The response to this question was binary (0 = no, 1 = yes).

### 2.4 Statistical analyses

Statistical analyses were done in STATA version 13. In the first place, descriptive statistics (frequency and percent distributions) were analyzed to describe the sample. Secondly, we performed statistical tests of associations between demographic, socio-economics, and health factors, and being sick in the past 30 days using chi-square tests. The level of statistical significance was set at 95% confidence (*p* = 0.05). Finally, we did a full binary logistic regression model to predict the factors associated with ill health or being sick in the past 30 days among older people in Uganda. We weighted the analysis (using survey (svy) commands) to account for the survey design including clustering, and stratification.

## 3 Results

### 3.1 Descriptive characteristics

Table 1 presents the descriptive characteristics of older persons stratified by gender. Overall, older women were slightly more numerous than men were (52% vs 48%). Majority of older people were aged 50–59 years (45%). The mean age was for both men and women was 62 years (minimum = 50, maximum = 98 and standard deviation = 11 years; results not presented in Table 1). The majority of the older people were from the eastern region (31%), lived in rural areas (90%), and with other people – not alone (91%). There were no significant differences between men and women by age (*p* = 0.56), region (*p* = 0.08), and living arrangement (*p* = 0.12).

Seven in ten (70%) older people headed households, over half (59%) were married and had no formal education (68%). Men, compared to women, were more likely to head household heads (88% vs 54%; *p* < 0.001); be in union or marriage (81% vs 38%; *p* < 0.01); and had better education levels (37% vs 27%; *p* < 0.01).

The majority (45%) of older persons (both men and women) were Catholics and were from non-poor households – spent >$1 a day (77%). A higher proportion of older persons depended on farming (61%) and wages (27%). However, a higher proportion of older women than men depended on remittances (17% vs 8%; *p* < 0.01). About one in five (22%) older persons had learnt a technical skill or trade. Four in ten (40%) of older people owned a bicycle. More older men's households than women owned bicycles (48% vs 32%; *p* < 0.01). There were no significant differences between men and women by poverty status (*p* = 0.48) and having learnt a technical skill or trade (*p* = 0.26).

About two in ten (23%) older persons reported at least one NCD (diabetes, heart diseases and high blood pressure) and a third (33%) had a disability. A higher proportion of women than men reported having an NCD (30% vs 16%; *p* < 0.01) or a disability (38% vs 28%; *p* < 0.01). Overall, six in ten (62%) older persons had been sick in the last 30 days. A higher proportion of women than men were sick in the last 30 days (67% vs 56%; *p* < 0.01).

### 3.2 Association between self-reported ill health and demographic and socio-economic factors

Table 2 presents chi-square test results for association between self-reported ill health and socio-economic, demographic and health factors, stratified by gender. Surprisingly, education and poverty status consistently had no association with self-reported ill health among older men, women, and all older persons.

Among all older persons, the prevalence of self-reported ill health was highest among women (67%; *p* < 0.001), oldest old – age 80+ (73%; *p* < 0.001), rural residents (63%; *p* < 0.045), those living alone (73%; *p* = 0.001) and the widowed (68%; *p* = 0.001). In addition, self-reported ill health was highest among Muslims (68%; *p* = 0.002), those who depended on remittances (75%; *p* < 0.001), had a technical skill (66%; *p* < 0.041), did not own a bicycle (65%; *p* < 0.004), reported NCDs (75%; *p* < 0.001) and had a disability (79%; *p* < 0.001). However, self-reported ill health did not vary significantly by region (*p* = 0.083), headship status (*p* = 0.051).

Among men, the prevalence of self-reported ill health was highest among those aged 70–79 years (71%; *p* < 0.001), those living alone (67%; *p* = 0.02), those who headed households (59%; *p* < 0.001) and those without a partner – separated or divorced or never married (66%; *p* = 0.05). Self-reported ill health was highest among men who were Muslims (62%; *p* = 0.014), did not own a bicycle (60%; *p* < 0.02), reported NCDs (65%; *p* = 0.02) and had a disability (77%; *p* < 0.001). However, self-reported ill health among men did not vary significantly by region (*p* = 0.662), residence (*p* = 0.996), source of earnings (*p* = 0.146), having learnt a technical skill (*p* = 0.353), education and poverty status.

Self-reported ill health among women was highest among the oldest old (76%; *p* = 0.01), those from eastern region (75%; *p* = 0.03), rural residents (69%; *p* < 0.035), those living alone (80%; *p* = 0.011), and headed households (71%; *p* = 0.01). Self-reported ill health among women was highest among those who depended on remittances (78%; *p* < 0.001), had a technical skill (73%; *p* < 0.032), reported NCDs (79%; *p* < 0.001) and had a disability (80%; *p* < 0.001). However, self-reported ill health among women did not vary significantly by marital status (*p* = 0.545), education (*p* = 0.569), religion (*p* = 0.104), poverty status (*p* = 0.09), and bicycle ownership (0.447). The prevalence of self-reported ill health was slightly higher among older women than that of men, on several demographic and socio-economic variables (Table 2).

### 3.3 Multivariable results

Table 3 presents results of the multivariable logistic regression of self-reported ill health among older persons in Uganda, stratified by gender. Among all older persons, self-reported ill health was associated with advanced age, being a woman, eastern region, being household head, Catholic, reporting NCDs, and disability. Older persons age 70–79, compared to those aged 50–59, had increased odds (OR = 1.53, 95% CI: 1.14–2.05) of reporting ill health. Older people who resided in the eastern region, relative to central region, were more likely (OR = 1.41, 95% CI: 1.04–1.91) to report illness during the past 30 days preceding the survey. Household heads, compared to their relatives, had increased odds of reporting ill health. Compared to Catholics, Seventh Day Adventists (SDA) and other religions, had decreased (OR = 0.41, 95% CI: 0.24–0.72) odds of being sick in the last 30 days.

Self-reported NCDs increased the risk (OR = 1.69, 95% CI: 1.31–2.17) of self-reported ill health among older persons. Similarly, disabled older persons had a higher likelihood (OR = 2.51, 95% CI: 1.98–3.18) of reporting ill health compared to those who were not. Finally, older women had increased odds (OR = 1.71; 95% CI: 1.29–2.27) of reporting ill health, compared to older men.

Among older men alone, self-reported ill health was associated with advanced age, being household head, being Catholic, and being disabled. Among older women alone, self-reported ill health was associated with eastern region, being household head, depending on farming relative to wages, reporting NCDs and having a disability (see Table 3).

## 4 Discussion

The aim of this paper was to estimate the prevalence of self-reported ill health and its associated factors among older persons in Uganda. More than half (62%) of the older persons reported ill health in the last 30 days. The prevalence of ill health among older persons in Uganda was higher (62%) in comparison to 42% in South Africa (Gómez-Olivé et al., 2013) or 48% in Singapore (George et al., 2012). In Addition, this prevalence of poor SRH was higher than the national estimate of 43% in the UNHS survey (UBOS, 2010). Self-reported ill health among all older persons was associated with advanced age, eastern region, being household head, being catholic, self-reported NCDs, disability, and being a woman.

Advanced age was associated with poor health. Advancement in age is associated with reduced immune response against illnesses, increased mobility limitations, and NCDs that impede access to healthcare and non-communicable diseases (Phaswana-Mafuya, Peltzer, Chirinda, Musekiwa, et al., 2013; Ward & Schiller, 2013). In addition, physiological changes that inhibit intake of nutrients and the factors already highlighted, contribute to poor physical health (Phaswana-Mafuya, Peltzer, Chirinda, Kose, et al., 2013; Tomás et al., 2012; Wasiak et al., 2014). However, the effect of advanced age on self-reported ill health was significant for older men alone and all older persons but not for older women alone (in the final model). For older women alone, the effect of age was mediated by disability in the final model.

Self-reported ill health among all older persons was associated with eastern region. It is important to note that the prevalence of self-reported ill health was highest in this region among men (59%), women (75%) and all older persons (67%). This finding was consistent with the prevalence of ill health in the general population in the 2010 UNHS report, where 43% of Ugandans reported ill health and eastern region had the highest prevalence (51%). In addition, eastern region had the highest prevalence of ill health among children under five years in the UNHS survey. This was due to the poorest socio-economic status (poverty) compared to other regions (UBOS, 2010). On the other hand, there were no regional variations in self-reported ill health among older men.

Household headship among older persons is an indicator of vulnerability (Golaz & Rutaremwa, 2011). Other studies in Uganda have reported that older persons were household heads and caregivers of HIV and AIDS orphaned children (Nyirenda, Newell, et al., 2013; Scholten et al., 2011; Seeley, Dercon, & Barnett, 2010; Seeley, Wolff, Kabunga, Tumwekwase, & Grosskurth, 2009). Older people do not earn viable incomes and are supposed to be dependent, yet they shoulder economic and social/physical and emotional responsibilities as they co-reside with orphaned children. It is difficult for them to think about their health when they have young people under their care who are ill (Seeley, Kabunga, Tumwekwase, Wolff, & Grosskurth, 2008; Seeley et al., 2009). Access to proper health care in older age is a challenge for most older people, especially those with limited social or family support (Schafer, 2013; Seeley et al., 2009).

Religion was associated with self-reported ill health among men and all older persons. The Seventh Day Adventists (SDAs) were less likely to be ill during the 30 days preceding the survey, compared to Catholics. SDA older persons are highly devoted to their religion and this could explain their better health outcomes. Studies have reported that deeply religious persons tend to have restrictive diets and are most likely to be healthier in the long term (Ferraro & Koch, 1994; Yeager et al., 2006). Restrictive diets reduce the risk of NCDs associated with affluent dietary habits. A cross tabulation of poverty and religion indicated that SDAs had the highest (48%) proportion of poor older persons, compared to (<30%) other religions (results not presented). However, the influence of religion on self-reported health requires further research.

Earning wages was associated with self-reported ill health among older women only. Women who earn wages were less likely to report ill health. This is not surprising because earning wages indicated that someone was either still in working age (44% of the women and retirement age in Uganda is 60 years) or they are younger older persons. However, the effect of wage earnings was not significant for older men and all older persons. Low socio-economic status has been associated with poor self-reported health in Netherlands (Koster et al., 2006), South Africa (Alaba & Chola, 2013; Phaswana-Mafuya, Peltzer, Chirinda, Musekiwa, et al., 2013) and Bangladesh (Khanam et al., 2011b) and Korea (Park, Cho, & Jang, 2012).

As expected, non-communicable diseases (NCDs) were associated with self-reported ill health among women and all older persons. NCDs such as heart disease, diabetes and hypertension, lead to regular episodes of attack, which leads to frequent ill health among older persons. In addition, they weaken the immune system, which makes older persons susceptible to frequent illness episodes (Khanam et al., 2011a; Marengoni et al., 2008; Phaswana-Mafuya, Peltzer, Chirinda, Musekiwa, et al., 2013; Stelmach et al., 2004; Ward & Schiller, 2013). Surprisingly, self-reported NCDs were not significantly associated with self-reported ill health among older men.

Disability was also associated with ill health among older persons in Uganda. Forms of disability, particularly those that limit mobility, impede physical or spatial accessibility to health care facilities (Gómez-Olivé et al., 2013). A study from Angola (Tomás et al., 2012) and South Africa (Phaswana-Mafuya, Peltzer, Chirinda, Kose, et al., 2013) reported this finding.

Finally, older women reported ill health more than older men. Women's poor health status reflects their lifelong experience of poor socio-economic status (Nimako et al., 2013), discrimination, deprivation and neglect (Kakoli & Anoshua, 2008; Razzaque et al., 2010). This finding confirms findings from IN-DEPTH network data (Gómez-Olivé et al., 2010, 2013; Hirve et al., 2010; Mwanyangala et al., 2010b) and other developing countries (Kabir et al., 2003b), where women reported poorer health status than men (Keskinoglu, Giray, Pıcakcıefe, Bilgic, & Ucku, 2005). Differential reporting might account for gender difference in ill health between men and women. It is also possible that men's health problems are under-reported because men are not expected to acknowledge illness as readily as women do (Kabir et al., 2003a). In addition, scholars have argued that women are more sensitive and easily report health conditions than men (Kakoli & Anoshua, 2008; Razzaque et al., 2010). They also interface with the healthcare system as caregivers of their children, more often than men do during their lifetime (Gómez-Olivé et al., 2013). Women also suffer from debilitating conditions but not fatal ones and this explains the paradox of high morbidity and less mortality among them compared to the men (Minh, Byass, Chuc, & Wall, 2010). However, new evidence suggests that this gender disparity in health status does not emerge when acute and chronic conditions are measured (Krause, Liang, Jain, & Sugisawa, 1998).

### 4.1 Study limitations and strengths

From the data, it is hard to ascertain the type or kind of sickness, which older persons suffered from. A follow up question to those who were sick asked about two primary symptoms that they suffered from. The symptoms included the following: diarrhea, weight loss, fever, malaria, skin rash, weakness, severe headache, fainting, chills (feeling hot and cold), vomiting, cough, coughing blood, pain on passing urine, genital sores, mental disorder, abdominal pain, sore throat, difficulty breathing, burn, fracture, wound, and child birth related illness. From the categories of the two symptoms, it is hard to tell the disease suffered from. The categories presented in the questionnaire did not include non-communicable diseases yet some of the older persons had them.

Self-reported health is a subjective measure of health status. In some studies, self-reported health was validated by taking objective measurements (Phaswana-Mafuya, Peltzer, Chirinda, Kose, et al., 2013), which was not the case in the Uganda National and Household survey. Under-reporting of health conditions such as NCDs and disability could have led to inaccuracies in self-reported data. However, subjective measures have been applauded for being “good measures for complex health problems” (Phaswana-Mafuya, Peltzer, Chirinda, Kose, et al., 2013) and providing estimates where objective measures are scarce.

The cross sectional nature of the UNHS does not allow cause-effect investigations (Kakoli & Anoshua, 2008). This implies that there is a need for longitudinal data to ascertain the direction of relationships among the variables under investigation. Nonetheless, the findings provide benchmarks for follow-up studies.

However, the strength of this study is that its findings are based on a nationally representative sample and they illuminate the knowledge gap about the health status of older persons in Uganda. Such vital information is useful in national planning for health service delivery tailored to addressing the needs of older persons in the country. In addition, it is representative of all older persons in Uganda.

## 5 Conclusions

Self-reported ill health among all older persons was associated with advanced age, eastern region of residence, being a household head, being Catholic, self-reported non-communicable diseases (NCDs), being disabled and being a woman.

Inequalities in health status exist among older people in Uganda. Interventions to improve the health of older people need to target the oldest old, the eastern region, those who head households, report NCDs and those who are disabled.

The primary healthcare system needs to scale up its efforts in the prevention and management of NCDs in old age in Uganda. The Ministry of Health (MoH) should prioritize NCDs especially hypertension, diabetes and heart disease, which predisposes older people to other disabling conditions like stroke. This will reduce co-morbidities and mortality among older people especially men. In addition, the disabled older persons need special attention.

There is need for further research on the health of older persons using the World Health Organization (WHO) life course approach. In particular, we need specialized surveys on older people to cover a broad spectrum of aging and health issues, to explore the relationship between region of residence, religious affiliation, and self-reported ill health. In particular, longitudinal studies including qualitative methods would be ideal. Such studies would capture better estimates of NCDs and disability among older people for better planning, policy formulation, and program design by the MoH.

## Conflict of interest

There is no conflict of interest declared.

## Figures and Tables

**Table 1:** Distribution of older persons by demographic, socio-economics, health factors and self-reported ill health, stratified by gender in Uganda.

	Men		Women		All		
Variables	Number (*n*)	Percent (%)	Number (*n*)	Percent (%)	Number (*n*)	Percent (%)	*p*-value
Gender							
Men					1136	47.7	
Women					1246	52.3	

Age group							0.56
50–59	524	46.1	542	43.5	1066	44.7	
60–69	313	27.6	356	28.6	670	28.1	
70–79	206	18.1	228	18.3	433	18.2	
80+	94	8.2	120	9.6	213	9.0	

Region							0.08
Central	278	24.5	311	24.9	589	24.7	
Eastern	371	32.6	358	28.7	728	30.6	
Northern	216	19.1	253	20.3	470	19.7	
Western	271	23.8	324	26.0	595	25.0	

Place of residence							0.95
Rural	1032	90.8	1131	90.7	2162	90.8	
Urban	104	9.2	115	9.3	220	9.2	

Living alone							0.12
No	1022	90.0	1145	91.9	2167	91.0	
Yes	114	10.0	101	8.1	215	9.0	

Relationship to household head							<0.01
Head	995	87.6	669	53.7	1664	69.9	
Spouse	72	6.3	387	31.0	458	19.2	
Relative	69	6.1	191	15.3	260	10.9	

Marital status							<0.01
Married	919	80.9	477	38.3	1396	58.6	
Div/Sep/never married^a^	84	7.4	158	12.7	242	10.2	
Widowed	133	11.7	611	49.0	744	31.2	

Education level							<0.01
None	713	62.8	908	72.8	1621	68.0	
Primary	302	26.6	287	23.0	589	24.7	
Secondary+	121	10.7	51	4.1	172	7.2	

Total	1136	100.0	1246	100.0	2382	100	

Religion							0.01
Catholic	527	46.4	549	44.1	1076	45.2	
Anglican	398	35.0	448	36.0	846	35.5	
Muslim	110	9.7	96	7.7	206	8.7	
Pentecostal	56	4.9	108	8.7	164	6.9	
SDA & Others	45	4.0	44	3.6	90	3.8	

Poverty status							0.48
Non-poor	883	77.7	955	76.7	1838	77.2	
Poor	253	22.3	291	23.3	544	22.8	

Household source of earnings							<0.01
Farming	720	63.4	731	58.7	1451	60.9	
Wages	330	29.0	307	24.7	637	26.7	
Remittances	86	7.6	208	16.7	294	12.3	

Learnt a trade or technical skill							0.26
No	875	77.0	983	78.9	1858	78.0	
Yes	261	23.0	263	21.1	524	22.0	

Household owns bicycle							<0.01
No	590	51.9	846	67.9	1436	60.3	
Yes	546	48.1	400	32.1	946	39.7	

Reported NCDs (heart disease, hypertension or diabetes)							<0.01
No	949	83.6	878	70.4	1827	76.7	
Yes	187	16.4	368	29.6	555	23.3	

Disabled							<0.01
No	823	72.4	777	62.4	1600	67.2	
Yes	314	27.6	469	37.6	782	32.8	

Sick in last 30 days							
No	495	43.6	409	32.8	904	38.0	
Yes	641	56.4	837	67.2	1478	62.0	

Total	1136	100.0	1246	100.0	2382	100	

a Never married were only 3 cases.

**Table 2:** Association between self-reported health and demographic, socio-economics, health factors and self-reported ill health, stratified by gender in Uganda.

Variables	Men			Women			All		
	% Sick	Number (*n*)	*p*-value	% Sick	Number (*n*)	*p*-value	% Sick	Number (*n*)	*p*-value
Gender									<0.01
Women							67.2	1246	
Men							56.4	1136	

Age group			<0.01			0.01			<0.01
50–59	49.1	524		62.1	542		55.7	1066	
60–69	56.0	313		67.4	356		62.1	670	
70–79	70.5	206		74.1	228		72.4	433	
80+	67.9	94		76.2	120		72.6	213	

Region			0.66			0.03			0.08
Central	55.3	278		64.5	311		60.1	589	
Eastern	59.1	371		74.7	358		66.8	728	
Northern	56.9	216		64.7	253		61.1	470	
Western	53.4	271		63.4	324		58.9	595	

Place of residence			0.99			0.03			0.05
Rural	56.4	1032		68.5	1131		62.7	2162	
Urban	56.4	104		53.7	115		55.0	220	

Living alone			0.02			0.01			<0.01
No	55.2	1022		66.0	1145		60.9	2167	
Yes	67.0	114		80.0	101		73.1	215	

Relationship to household head			<0.01			0.01			0.05
Head	58.8	995		70.6	669		63.6	1664	
Spouse	31.5	72		65.9	387		60.5	458	
Relative	47.4	69		57.5	191		54.8	260	

Marital status			0.05			0.55			<0.01
Married	54.7	919		65.6	477		58.4	1396	
Div/Sep/never married^a^	65.6	84		65.2	158		65.4	242	
Widowed	62.7	133		68.9	611		67.7	744	

Education level			0.39			0.57			0.98
None	55.8	713		67.2	908		62.2	1621	
Primary	55.4	302		68.3	287		61.7	589	
Secondary+	62.5	121		60.2	51		61.8	172	

Religion			0.01			0.11			<0.01
Catholic	58.2	527		68.0	549		63.2	1076	
Anglican	56.0	398		66.1	448		61.4	846	
Muslim	62.2	110		74.5	96		67.9	206	
Pentecostal	53.3	56		67.6	108		62.7	164	
SDA & Others	29.7	45		49.6	44		39.6	90	

Poverty status			0.36			0.09			0.58
Non-poor	55.6	883		68.6	955		62.4	1838	
Poor	59.1	253		62.5	291		60.9	544	

Household earnings			0.15			<0.01			<0.01
Farming	55.5	720		68.2	731		61.9	1451	
Wages	55.5	330		57.5	307		56.5	637	
Remittances	67.6	86		77.7	208		74.8	294	

Learnt a trade or technical skill			0.35			0.03			<0.01
No	55.6	875		65.5	983		60.8	1858	
Yes	59.2	261		73.4	263		66.3	524	

Household owns bicycle			0.02			0.45			<0.01
No	59.9	590		68.0	846		64.7	1436	
Yes	52.6	546		65.4	400		58.0	946	

Reported an NCD (heart disease, hypertension or diabetes)			0.02			<0.01			<0.01
No	54.7	949		62.1	878		58.2	1827	
Yes	65.2	187		79.3	368		74.5	555	

Disabled			<0.01			<0.01			<0.01
No	48.8	823		59.4	777		53.9	1600	
Yes	76.5	314		80.0	469		78.6	782	

Total	56.4	1136		67.2	1246		62.0	2382	

a Never married were only 3 cases.

**Table 3:** Results of multivariable logistic regression of self-reported ill health on socio-economic, demographic and health-related factors among older people in Uganda.

Variables	Men		Women		All	
	Odds ratio	95% CI	Odds ratio	95% CI	Odds ratio	95% CI
Age group (rc = 50–59)						
60–69	1.14	[0.81–1.61]	1.05	[0.78–1.43]	1.12	[0.87–1.44]
70–79	1.84^**^	[1.25–2.71]	1.24	[0.81–1.90]	1.53^**^	[1.14–2.05]
80+	1.43	[0.78–2.60]	1.61	[0.88–2.95]	1.51	[0.96–2.37]
Region (rc = central)						
Eastern	1.27	[0.85–1.89]	1.86^**^	[1.18–2.92]	1.41^*^	[1.04–1.91]
Northern	1.16	[0.76–1.78]	1.24	[0.82–1.87]	1.15	[0.86–1.54]
Western	0.99	[0.62–1.56]	1.10	[0.68–1.78]	1.02	[0.73–1.43]
Place of residence (rc = rural)	1.10	[0.59–2.07]	0.66	[0.38–1.16]	0.83	[0.58–1.19]
Living alone (rc = live with others)	0.80	[0.43–1.46]	1.41	[0.78–2.57]	1.29	[0.88–1.90]
Relationship to household head (rc = head)						
Spouse	0.34^***^	[0.19–0.61]	0.91	[0.55–1.51]	0.73	[0.52–1.02]
Relative	0.41^**^	[0.21–0.78]	0.64^*^	[0.43–0.96]	0.62^**^	[0.44–0.85]
Marital status (rc = married)						
Divorced/separated/never married	1.41	[0.74–2.68]	0.90	[0.48–1.66]	0.88	[0.59–1.31]
Widowed	1.33	[0.80–2.22]	0.88	[0.52–1.48]	0.82	[0.60–1.13]
Religion (rc = Catholic)						
Anglican	0.88	[0.63–1.25]	0.83	[0.60–1.16]	0.86	[0.67–1.11]
Muslim	1.14	[0.68–1.92]	1.02	[0.59–1.76]	1.07	[0.73–1.58]
Pentecostal	0.92	[0.49–1.73]	0.91	[0.55–1.50]	0.88	[0.60–1.30]
SDA and others	0.32^**^	[0.15–0.66]	0.59	[0.29–1.19]	0.41^**^	[0.24–0.72]
Household earnings (rc = farming)						
Wages	1.05	[0.74–1.48]	0.69^*^	[0.50–0.97]	0.88	[0.69–1.12]
Remittances	0.98	[0.57–1.68]	1.21	[0.79–1.86]	1.13	[0.80–1.59]
Learnt a trade or technical skill (rc = no)	1.14	[0.82–1.59]	1.34	[0.91–1.98]	1.17	[0.93–1.49]
Household owns bicycle (rc = no)	0.77	[0.59–1.00]	1.03	[0.75–1.42]	0.90	[0.73–1.11]
Self-reported NCDs (rc = no)	1.33	[0.92–1.93]	2.06^***^	[1.46–2.90]	1.69^***^	[1.31–2.17]
Disabled (rc = no)	3.06^***^	[2.18–4.31]	2.17^***^	[1.59–2.96]	2.51^***^	[1.98–3.18]
Gender (rc = men)					1.71***	[1.29–2.27]

Observations	1136		1246		2382	
Link test hat (*p*-value)	<0.001		<0.001		<0.001	
Link test hat-squared (*p*-value)	0.76		0.28		0.55	
Goodness of fit test (*p*-value)	0.83		0.95		0.74	
Model *p*-value	<0.001		<0.001		<0.001	

rc, reference category.
